# Limited Propagation of SARS-CoV-2 among Children in a Childcare Center, Canada, 2021

**DOI:** 10.3201/eid2801.211811

**Published:** 2022-01

**Authors:** Toni Li, Kieran Moore, Lindsay Bowthorpe, Julie Sousa, T. Hugh Guan

**Affiliations:** Kingston, Frontenac, Lennox & Addington Public Health, Kingston, Ontario, Canada (T. Li, K. Moore, L. Bowthorpe, J. Sousa, T.H. Guan);; Queen’s University, Kingston (T. Li, K. Moore, L. Bowthorpe, T.H. Guan);; Ministry of Health of Ontario, Toronto, Ontario, Canada (K. Moore)

**Keywords:** COVID-19, SARS-CoV-2, COVID-19 testing, severe acute respiratory syndrome coronavirus 2, coronavirus disease, disease outbreaks, infection control, public health, child day care centers, contact tracing, patient isolation, respiratory infections, viruses, zoonoses

## Abstract

An outbreak of severe acute respiratory syndrome coronavirus 2 with no definitive source and potential exposure to variants of concern was declared at a childcare center in Ontario, Canada, in March 2021. We developed a robust outbreak management approach to detect, contain, and interrupt this outbreak and limit propagation among children.

On March 1, 2021, an infant enrolled at a childcare center in the Kingston, Frontenac, Lennox, and Addington region in Ontario, Canada tested positive for severe acute respiratory syndrome coronavirus 2 (SARS-CoV-2); however, no acquisition source was identified. The next day, another 7 children and staff at the facility tested positive, and an outbreak was declared. 

We immediately searched for potential transmission events and deployed a public health inspector and nurse team. The infant had last attended the childcare center >3 days before symptom onset, beyond the 48-hour window for exposure risk according to standard guidance (*1*). Furthermore, the assessment team identified no travel, occupational, or other contact risks. Out of an abundance of caution, we extended the period of communicability (POC) from 48 to 96 hours, which defined the childcare center as an outbreak setting. We identified staff who had recently traveled to regions with high proportions of SARS-CoV-2 variants of concern (VOC). We were concerned that the increased transmissibility and virulence of a potential VOC outbreak in a childcare center could rapidly spread through the community, given recent studies demonstrating SARS-CoV-2 infection and transmission among children (*2*,*3*).

Case investigators gathered symptom profiles, onset dates, detailed exposure histories, risk factors, and contacts. Because they did not meet early vaccine eligibility criteria, none of the persons had been vaccinated. Because of concern about VOCs, we applied the 96-hour POC to all case-patients and high-risk contacts. Case-patients were required to immediately isolate for 10 days under active monitoring. We advised all high-risk contacts and their household contacts to quarantine for 14 days. As a precaution, we initiated contact tracing before receiving laboratory results for high-risk contacts in whom COVID-19-associated symptoms developed. We requested that all close contacts be tested 3 times while in quarantine: on day 0 and during days 5–7 and 10–12. To be discharged, we required contacts to test negative on days 10–12 or, if having a positive or incomplete test, to quarantine for 10 additional days before retesting. The local Public Health Ontario laboratory conducted real-time reverse transcription PCR testing using the cobas 6800/8800 assay (Roche Molecular Diagnostics, https://diagnostics.roche.com) or a laboratory-developed test (Public Health Ontario, https://publichealthontario.ca) (*4*,*5*). Testing turnaround time was <24 hours, and positive samples were tested for N501Y and E484K mutations.

A total of 21 SARS-CoV-2 cases were associated with this outbreak during March 1–23, 2021 (Appendix): 14 (67%) through direct exposure at daycare and 7 (33%) through secondary transmission (Figure). Average affected age was 22.5 years (range 19 months–68 years); similar proportions of female (11/21) and male (10/21) persons were affected.

**Figure:**
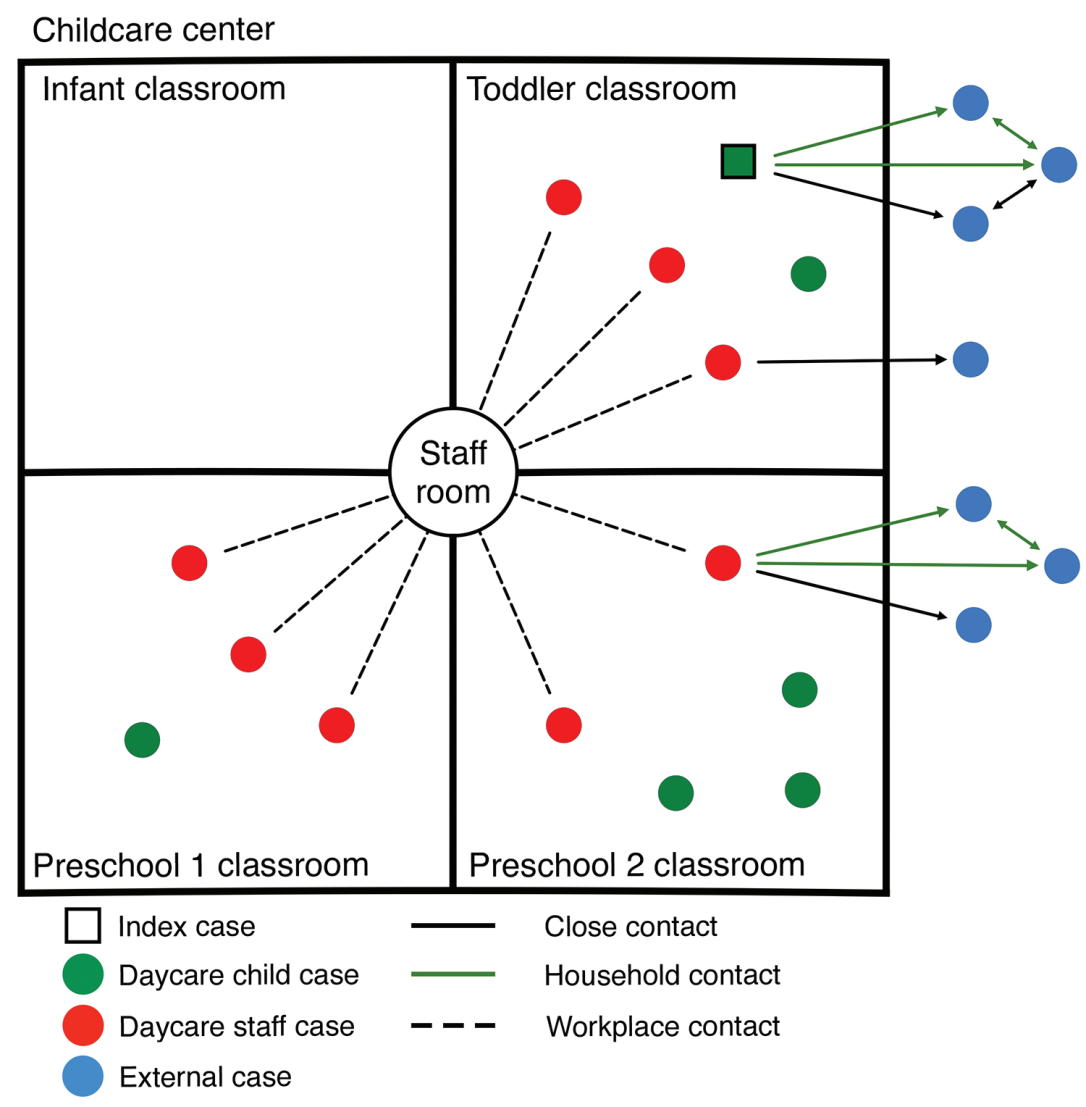
Social network analysis of a COVID-19 outbreak in a childcare center in Ontario, Canada, March 1–23, 2021. The facility had 1 common staff room and 4 physically separated classroom cohorts: infant (6–18 months of age), toddler (18 months–2.5 years of age), and preschool classes 1 and 2 (both 2.5–5 years of age), excluding adult staff.

For the first generation, the staff attack rate, 47% (8/17), was >4 times higher than the child attack rate, 11% (6/53) (Table) and higher in every classroom with positive cases, aligning with increased SARS-CoV-2 susceptibility and transmission reported among adults compared with children (*6*–*8*). Of note, we observed no cases or transmission among nonmobile infants, who remained in assigned cribs in a separate classroom, or their caregivers.

**Table:** Severe acute respiratory syndrome coronavirus 2 cases and attack rates staff and children during outbreak associated with childcare center, Ontario, Canada, March 1–23, 2021

Category	Infant classroom	Toddler classroom	Preschool 1 classroom	Preschool 2 classroom	Total
Children					
Total no.	6	12	20	15	53
No. cases	0	2	1	3	6
Attack rate, %	0	17	5	20	11
Staff					
Total no.	3	3	8	3	17
No. cases	0	3	3	2	8
Attack rate, %	0	100	38	67	47

We observed proper personal protective equipment, hand hygiene, and cleaning protocols. However, we identified staff breakrooms as high-risk settings because of reduced physical distancing between different staff cohorts and long-term unmasking during meals. Furthermore, some staff were identified as coming to work with COVID-19–associated symptoms which, when combined with high risk for staff exposure, emphasizes the continued importance of careful screening at work and requiring isolation and retesting after a positive test (*9*). Staff must also be vigilant in adhering to physical distancing and infection prevention and control guidelines (https://ipac-canada.org) when socializing outside of the workplace.

Although we identified no definitive acquisition source or transmission incidents, our robust outbreak management approach enabled detection, containment, and interruption of this outbreak with limited propagation among children (Appendix Figure). Extending the POC from 48 to 96 hours broadened our capacity to identify both exposure risks and VOC risk from staff travel. Immediate lockdown of facilities and rapid isolation and quarantine guidance reduced further transmission. The 3-stage testing strategy and short testing turnaround times helped us identify 5 persons who tested positive after initially testing negative (3 on days 5–7 and 2 on days 10–12 of isolation) who might otherwise have further transmitted the virus. Early identification of contacts from second-generation cases and rapid closure, isolation, and testing of other at-risk locations prevented third-generation spread; there was no reported transmission in other workplaces, schools, or the community. We detected no VOCs and presumed this outbreak to involve wild-type strain. No case-patients required hospitalization or died during this outbreak. Our findings show that an aggressive testing protocol, strong collaboration with persons in the outbreak setting, and concentric circle quarantining of contacts were crucial to successfully managing a potential VOC outbreak, particularly when no specific acquisition sources or exposure risks were known.

Appendix Additional information about a severe acute respiratory syndrome coronavirus disease outbreak in a childcare center, Canada, 2021.
