# Supplementary material for: Limited Propagation of SARS-CoV-2 among Children in a Childcare Center, Canada, 2021
Source: Emerg Infect Dis. 2022 Jan;28(1):259–62. doi: 10.3201/eid2801.211811 (PMC8714234; doi:10.3201/eid2801.211811)

Article DOI: https://doi.org/10.3201/eid2801.211811

Limited Propagation of SARS-CoV-2 among Children in a Childcare Center, Canada, 2021

Appendix

Appendix Table. Summary of outbreak management approach for a suspected variant of concern COVID-19 outbreak in a childcare center, Canada, March 1–23, 2021*

Control strategy	Confirmed case	HR contact	HHC of HR contact
Isolation/quarantine period	10 d if symptoms resolved	14 d	14 d or until HR contact tested negative days 10–12; if positive, HHC redesignated HR contact
Testing	Day 0, day 5–7, days 10–12	Day 0, day 5–7 (when possible), days 10–12	None unless symptomatic or HR contact tested positive
Day 10–12 test positive or not completed	Discharged after required isolation completed	Quarantine extended by 10 d	NA
Period of communicability	96 h	96 h	NA
Contact notification	Immediately following confirmation of outbreak	If symptoms developed, initiated before lab result receipt	NA

*COVID-19, coronavirus disease; HR, high risk; HHC, household contact; NA, not applicable; VOC, variant of concern

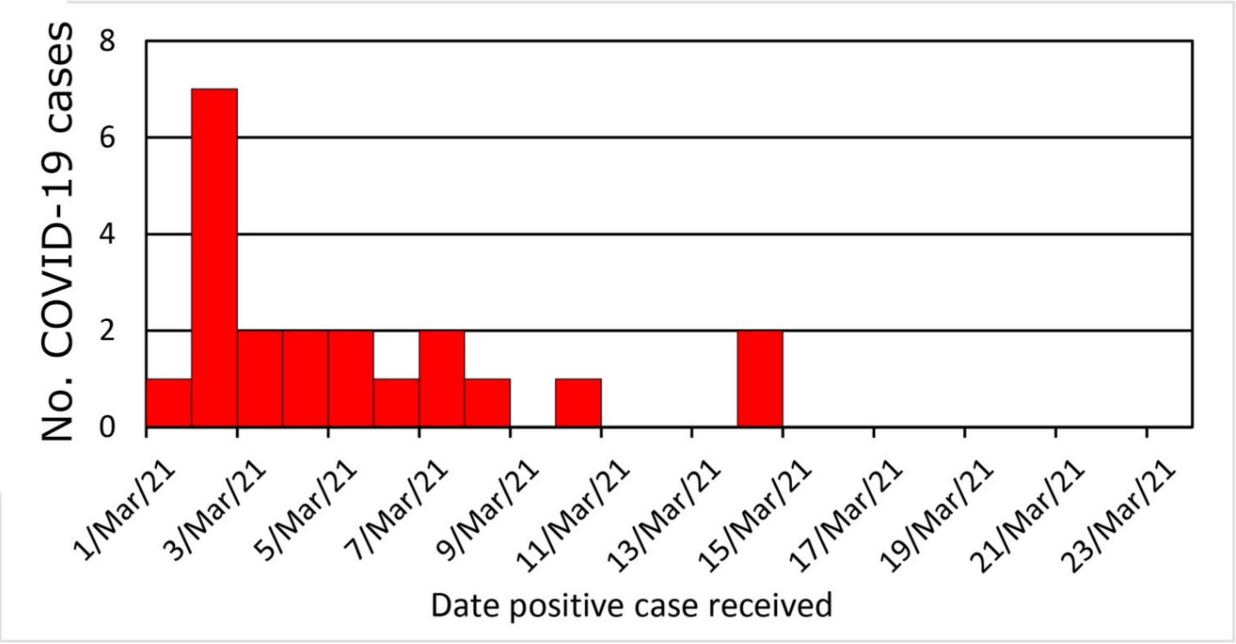

Supplement: Appendix — Additional information about a severe acute respiratory syndrome coronavirus disease outbreak in a childcare center, Canada, 2021. [file 21-1811-Techapp-s1.pdf]
